# In commemoration of Prof. M.R. Warburg and of his contribution to terrestrial Isopod biology (31 May 1931, Berlin–9 February 2014, Haifa)

**DOI:** 10.3897/zookeys.515.9909

**Published:** 2015-07-30

**Authors:** Elisabeth Hornung

**Affiliations:** 1 Szent István University, Faculty of Veterinary Science, Institute for Biology, H-1077 Budapest, Rottenbiller str. 50., Hungary

Our scientific community has lost one of its prominent members: Prof. Michael R. Warburg (MRW) who passed away on the 9th of February, 2014 in Haifa. The organisers of the 9th Symposium on the Biology of Terrestrial Isopods, held in Poitiers, France, decided to dedicate the meeting and this special issue to his memory. Prof. Warburg was a highly regarded member of our community, a ‘spiritual sponsor’ of isopod research, a passionate isopodologist himself, and a mentor to many students and young researchers.

## M.R. Warburg and the symposia series

The foreword of the 1st Symposium on the Biology of Terrestrial Isopods volume (London, 7–8^th^ July 1983) (Sutton and Holdich 1984) states that MRW was ’the father’ of this series of symposia as he originally suggested bringing together “*people with an interest in terrestrial isopods and try to present as wide a range of papers as possible*”. The idea proved to be a great success. The first meeting was followed by several others at intervals of 3–5 years. MRW also initiated a meeting in Vancouver, Canada, in 1992. This symposium, under the umbrella of the American Society for Zoologists’ regular congress, was organized by Prof. M.A. Alikhan (Sudbury, Ontario, Canada). MRW also lent his support to a smaller workshop in Hungary (Gödöllő) in 1991 connected to the 4th European Congress of Entomology. E. Hornung and K. Szlavecz organized the workshop, including a round table discussion that resulted in a summary on the trends and methods in terrestrial isopod ecology (Hornung et al. 1992). The last meeting MRW participated in was in 1997, in Haifa Israel. This symposium was jointly organized by MRW himself and E. Hornung. The latest meeting attracted the isopodologists’ crowd to Poitiers last summer (26–30. June, 2014). This meeting was a worthy tribute to Professor Michael Warburg’s memory.

## Scientific career and personal life of M.R. Warburg

Michael R. Warburg was born in Berlin, Germany in 1931. His family left the country in 1934 due to the Nazi threat. They moved first to London and, shortly after, to Haifa, then under British mandate, in Palestine. His reminiscences start in that period, from when he was five years old. MRW himself divided his memoir into periods which I have followed in this tribute (see also Hornung and Warburg 2014).

### Childhood (1936–1946)

The family recalls that “*he was always collecting animals, and had all sorts of self-made cages for reptiles and other animals, which he kept on the roof of the house. Though Haifa was a city, in those days you didn’t need to go far in order to reach the natural environment, it was right there, and he used to spend most of his time outside*”. He himself mentions in his memoir that “*Already as a young boy, I used to walk with my late father, an ardent naturalist at heart, though to his regret not by profession, in the fields and woods on Mt. Carmel where we lived*”. He often mentioned his great walks while bird watching with his father on the marshy plains (now housing estates) around Mt. Carmel. He dedicated his book on isopods (Warburg 1993) to his father and uncle: “*In memory of my late father*, Sigmund, *and uncle*, Edgar, *who both influenced in different ways my approach to nature*”.

### Graduate studies at the Hebrew University (1950–1954)

His university studies started with some difficulties, as he failed the preliminary biology exams and had to start his first semester in the Faculty of Mathematics. Still, from time to time he was able to attend biology lectures. During the second semester he was allowed to choose a few laboratory practicals and finally, at the end of the second year he transferred to major in biology. In his third year he received an assistantship on the entomology course. He started to work on his MSc thesis under Dr A. Zuckerman on the life cycle of *Trypanosoma lewisii*, a blood parasite of rodents. His attempts to locate the parasites were not successful, so he moved to a different research project: producing a serum against *Plasmodium berghei*, a blood sporozoan lethal to hamsters. With time-consuming, diligent work (“*half a year’s work which I did working days and nights*”) he successfully finished the project in 1954, receiving the grade ‘very good’. In 1955, he reached two milestones in his life: he published his first paper, and married Hava, his wife for the next 59 years.

### Teachers Seminary Oranim (1955–1956)

He was appointed to a position in Oranim, in the Teachers’ Seminary. “*Throughout the entire period I have conducted field trips in the country and have collected isopods. My main difficulty was how to identify them*.” He made every effort to familiarize himself with isopod identification using Vandel’s (1960, 1962) papers on isopod taxonomy. He also sought help from two scientists at the Vienna Naturhistorisches Museum, Dr. F. Strouhal, and Dr. K. Schmölzer, experts on the western Mediterranean isopod fauna. MRW himself never became a taxonomist, instead, he was more interested in the physiology, morphology and ecology of isopods.

While teaching in Oranim, he came across Edney’s (1954) review paper on woodlice and their land habitat. He decided to find an university where he could conduct research in this topic for his Ph.D. thesis. In 1956 he was accepted into the graduate program at Yale University, in the USA.

### Graduate studies at Yale University, New Haven, Connecticut, US (1956–1960)

In his application he outlined three research plans and “*Yale University offered both a fellowship and the choice of one of the three subjects for research towards a Ph.D. suggested by me*…”. He decided on the ‘The ecological, behavioral and physiological adaptations of terrestrial isopods’ with Prof. G.E. Hutchinson as his supervisor. First, he started with physiological and behavioral studies in the lab on local species, then switched to work on a desert species, *Venezillo arizonicus*. The research focused on adaptations to different microhabitats and microclimatic conditions such as relative humidity and temperature, using thermo-hygrograms. He quantified the behavioral responses of isopods to such conditions using a choice chamber / thermo-preference apparatus. He concluded that the interaction of three environmental factors, temperature, humidity and light, explain the majority of microhabitat choices of terrestrial isopods. He published his results on physiological ecology, specifically on water balance (evaporative water loss) and thermal balance of isopods in four papers (nos: 1-4).

After the completion of his Ph.D. in 1960, he returned to Israel and began looking for a job. It was not easy but finally he got an one-year position at the Tel Aviv University.

### Tel-Aviv University (1960–1961)

MRW had limited facilities there, but he was able to conduct extensive field work, collecting isopods in all parts of the country. Field work was an essential part MRW’s professional life. He summarized 80 years of isopod collecting efforts, 40 years of which was his own, in a review paper in 2007 (no. 45). Collecting sites in over 600 localities were visited approximately 900 times, resulting in a total of 41 species records for Israel. The collection is now owned by the university in Tel Aviv.

### University of South Australia (1962–1964)

In 1962 MRW was awarded a Senior Research Fellowship at the Zoology Department, University of Adelaide, South Australia. Although his research there, under the supervision of Prof. H.G. Andrewartha, focused on the ecology of *Tiliqua rugosa*, a large skink, he managed to study woodlice as well. He conducted experimental work on evaporative water loss and thermal balance of isopods under controlled conditions. This fellowship resulted in eight papers, two of them on terrestrial isopods. (Nos 5-6).

He returned back to Israel in 1964 and, after several temporary jobs, he received a position at the Israel Institute for Biological Research (Ness Ziona, near Tel Aviv).

### Israel Institute for Biological Research (1965–1972)

Here, he studied the cave tick, *Ornithodorus tholozani*, specifically its neurosecretory cells, reproduction and ecology. He published 11 papers on this topic. He learned techniques in neurosecretion at Sheffield University, under Prof. K. Highnam and in Paris, under Prof. M. Gabe, and utilized this knowledge in isopod research (No 10). He continued collecting isopods extensively. This time he had professional help from Dr. H. Schmalfuss (Natural History Museum, Stuttgart, Germany), who both verified MRW’s identifications and identified several new species, naming one of them after MRW: *Chaetophiloscia warburgi* Schmalfuss, 1991.

### TECHNION (1972–2013)

**Figure 1.**
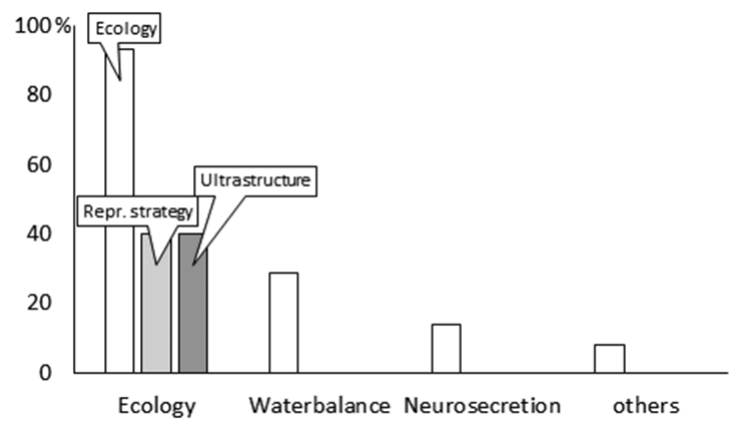
The distribution of MR Warburg’s papers on isopods by topic.

He spent his last 40 years at the Israeli Institute for Technology (TECHNION), Haifa. His papers published during this period can be grouped mainly into ecological, ecophysiological and behavioral topics (Fig. 1). These themes overlap in his publications as he used diverse methods and approaches to describe the phenomena in question.

One research focus was species diversity and distribution mainly in the northern part of the Mediterranean region, where several different localities differ in plant and stone coverage and in climatic conditions. MRW was also interested in the intra-habitat dispersion of woodlice, specifically how the number and distribution of potential sheltering sites influenced the presence of isopods in different types of habitats.

The second field of MRW’s interest was population and life history. He conducted research on the population structure, sex rate, life history and reproductive strategies of isopods. The latter included detailed studies of the structure of the female reproductive system and structure of the brood-pouch.

Behavioral studies were focused on responses of woodlice to temperature, light and humidity and resulted in two papers (nos: 1, 7).

In one of MRW’s papers (no 2) he reported on osmolarity of isopods under different environmental conditions.

Later, during his sabbaticals he revisited his favourite places in the US and Australia and, in collaboration with colleagues such as Prof. M.A. Alikhan in Ontario, Canada; Prof. C. Crawford in New Mexico, US, and Prof. P. Greenaway, New South Wales, Australia, conducted new research on isopod ecology. In Haifa he kindly hosted several isopodologists, including the author and collaborated internationally in laboratory and field projects.

## M.R. Warburg’s scientific achievements

**Figure 2.**
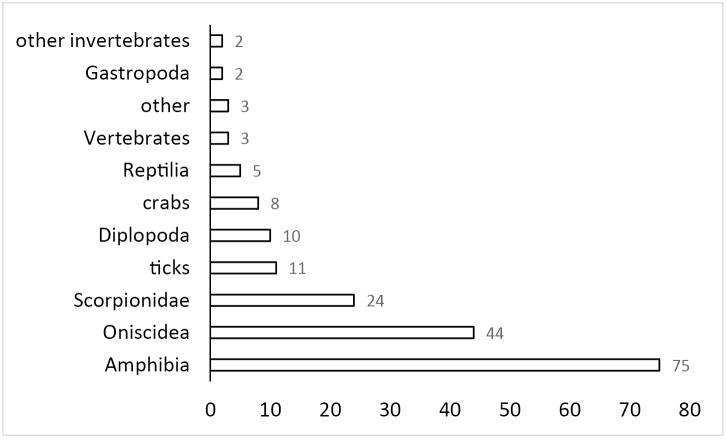
Distribution of published papers by animal groups.

**Figure 3.**
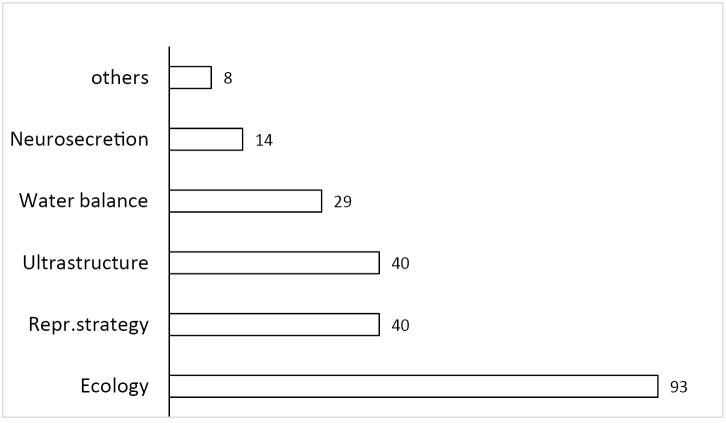
Percentage of MRW’s publications falling into different topics. (One paper may cover several subjects.)

During his scientific career M.R. Warburg studied a broad range of animal taxa such as ticks, scorpions, amphibians, and reptiles in addition to terrestrial isopods (Fig. 2). All these diverse projects fit within the disciplines of species diversity, distribution, ecophysiology, reproductive systems and strategies (Fig. 3). He published more than 180 papers, over 75 abstracts, 2 books and was the co-editor of the 4th Symposium on the Biology of Terrestrial Isopods volume.

Even after his retirement MRW continued to be scientifically active. As Professor Emeritus, he focused on summarizing his results and sharing them with the scientific community. In the past 13 years he kept publishing; fourteen of these papers are (partial) reviews on his favorite taxa.

He is survived by his wife, Hava (a biology teacher) his son Ittai, his daughters Meirav and Sharon and 11 grandchildren (photo 13).

**Figure:**
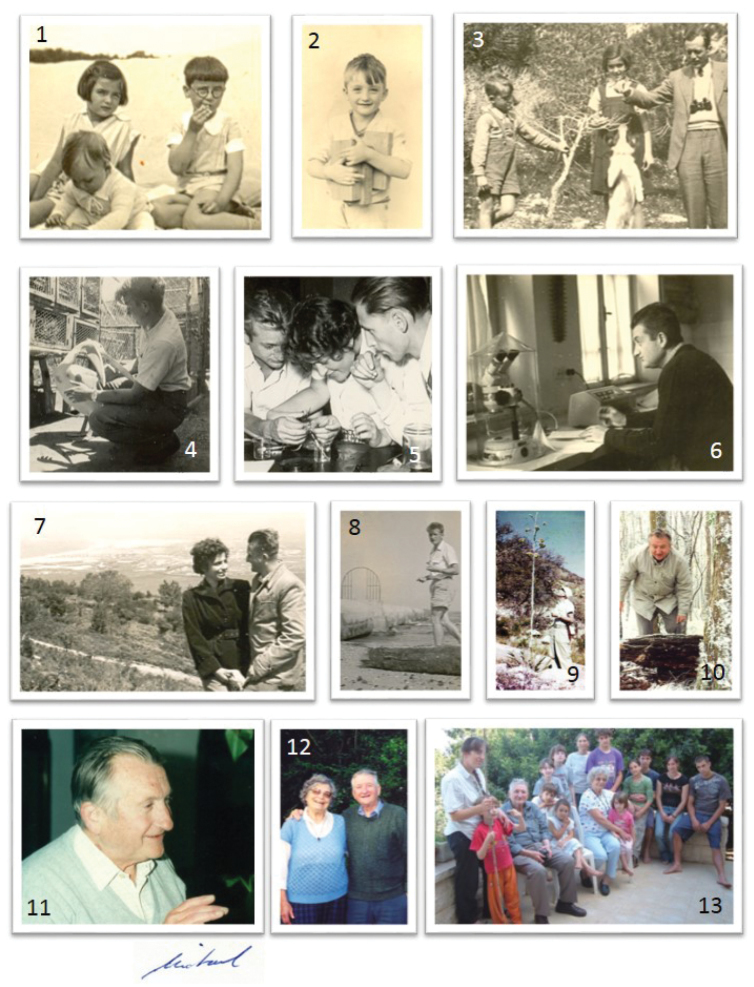
**1** In the company of his brother (Gaby) and sister (Hanne), 1932 **2** 1936 (5 years old) when his memoir started **3** With his father and sister on the field **4** Cages everywhere... With a pelican (1952) **5** Physiology lab (1954) **6** Israel Institute for Biological Research (1965) **7** With Hava Warburg in 1956 (marrige 1955) **8** On a zoology excursion, 1954 **9** Santa Rita experimental station, USA, 1957 **10** 1990: Searching for Isopods. Sabbatical in New South Wales **11** 1997, Haifa Symposium, Farewell party **12** 1982: In New Zealand with Hava during sabbatical **13** The last family photo in 2010

## References

[B1] Edney EB (1954) Woodlice and the land habitat. Biological Reviews 29(2): 185–219. doi: 10.1111/j.1469-185X.1954.tb00595.x

[B2] Hornung E Warburg S (2014) Tribute to Michael R. Warburg (31 May 1931–9 February 2014). Crustaceana 87(11-12): 1453–1460. doi: 10.1163/15685403-00003360

[B3] Hornung E Szlavecz K Warburg MR (1992) Trends and methods in terrestrial isopod ecology: a round-table discussion. Proc. ECE/XIII, SEEC, Gödöllő, 2 Hungarian Natural History Museum, Budapest, 747–750.

[B4] Sutton SL Holdich DM (1984) The Biology of terrestrial isopods: the proceedings of a symposium held at the Zoological Society of London on 7th and 8th of July 1983. Published for the Zoological Society of London by Clarendon Press, 518 pp.

[B5] Vandel A (1960) Faune de France, vol. 64 Isopodes terrestres (premiere partie). Paris, 416 pp.

[B6] Vandel A (1962) Faune de France, 66 Isopodes terrestres (deuxieme partie). Paris, 417–931.

[B7] Warburg MR (1955) An attempt to produce a specific serum against *Plasmodium berghei* in the rabbit. Bulletin of the Research Council of Israel 5(B): 144–147.

[B8] Warburg MR (1993) Evolutionary Biology of Land Isopods. Springer-Verlag, Berlin Heidelberg, 159 pp.

[1] Warburg MR (1964) The response of isopods towards temperature, humidity and light. Animal Behaviour 12: 175–186. doi: 10.1016/0003-3472(64)90119-8

[2] Warburg MR (1965a) Water relation and internal body temperature of isopods from mesic and xeric habitats. Physiological Zoology 38: 99–109. http://www.jstor.org/stable/30152347

[3] Warburg MR (1965b) The microclimate in the habitats of two isopod species in southern Arizona. The American Midland Naturalist 73: 363–375. doi: 10.2307/2423460

[4] Warburg MR (1965c) The evolutionary significance of the ecological niche. Oikos 16: 205–213. doi: 10.2307/3564874, http://www.jstor.org/stable/3564874

[5] Warburg MR (1965d) The evaporative water loss of three isopods from semi-arid habitats in South Australia. Crustaceana 9: 302–308. doi: 10.1163/156854065X00073

[6] Warburg MR (1968a) Simultaneous measurement of body temperature and weight loss in isopods. Crustaceana 14: 39–44. doi: 10.1163/156854068X01150

[7] Warburg MR (1968b) Behavioral adaptations of terrestrial isopods. American Zoologist 8: 545–559, 599–601. doi: 10.1093/icb/8.3.545

[8] Warburg MR Berkovitz K (1978a) Thermal effects on photoreaction of the oak-woodland pillbug *Armadillo officinalis* (Isopoda; Oniscoidea), at different humidities. Journal of Thermal Biology 3: 75–78. doi: 10.1016/0306-4565(78)90041-4

[9] Warburg MR Berkovitz K (1978b) Hygroreaction of normal and desiccated *Armadillo officinalis* isopods. Entomologia Experimentalis et Applicata 24: 55–64. doi: 10.1111/j.1570-7458.1978.tb02756.x

[10] Warburg MR Rosenberg M (1978) Neurosecretory cells in the brain of *Porcellio obsoletus* (Isopoda: Oniscoidea). International Journal of Insect Morphology & Embryology 7: 195–204. doi: 10.1016/0020-7322(78)90002-8

[11] Warburg MR Rankevich D Chasanmus K (1978) Isopod species diversity and community structure in mesic and xeric habitats of the Mediterranean region. Journal of Arid Environments 1: 157–163.

[12] Warburg MR Linsenmair KE Bercovitz K (1984) The effect of climate on the distribution and abundance of isopods. Symposia of the Zoological Society of London 53: 339–367. urn:nbn:de:bvb:20-opus-44473

[13] Hadley NF Warburg MR (1986) Water loss in three species of xeric -adapted isopods: correlations with cuticular lipids. Comparative Biochemistry and Physiology 85(A): 669–672.3791966

[14] Warburg MR (1987) Haemolymph osmolality, ion concentration and the distribution of water in body compartments of terrestrial isopods under different ambient conditions. Comparative Biochemistry and Physiology 86(A): 433–437. doi: 10.1016/0300-9629(87)90520-2

[15] Warburg MR (1987) Isopods and their terrestrial environment. Advances in Ecological Research 17: 187–242. doi: 10.1016/s0065-2504(08)60246-9

[16] Warburg MR Rosenberg M (1989) Ultracytochemical identification of Na+, K+ -ATPase activity in the isopodan hindgut epithelium. Journal of Crustacean Biology 9: 525–528. doi: 10.2307/1548584

[17] Warburg MR (1989) The role of water in the life of terrestrial isopods. Monitore Zoologico Italaliano Monographs (N.S.) 4: 285–304.

[18] Warburg MR (1991) Reproductive patterns in oniscid isopods. In: Juchault P Mocquard JP (Eds) ‘Biology of Terrestrial Isopods’, 3rd International Symposium, Poitiers Universite de Poitiers Press, 131–137.

[19] Warburg MR Cohen N (1991) Reproductive pattern, allocation and potential in a semelparous isopod from the Mediterranean region of Israel. Journal of Crustacean Biology 11: 368–374. doi: 10.2307/1548463

[20] Warburg MR (1992a) Life history patterns of terrestrial isopods from mesic habitats in the temperate region of northern Israel (Isopoda: Porcellionidae, Armadillidae). Studia on Neotropical Fauna & Environment 27: 155–165. doi: 10.1080/01650529209360875

[21] Warburg MR (1992b) Reproductive patterns in three isopod species from the Negev desert. Journal of Arid Environments 22: 73–85.

[22] Warburg MR Cohen N (1992a) Reproductive pattern, allocation and potential of an iteroparous isopod from a xeric habitat in the Mediterranean region. Journal of Arid Environments 22: 161–171.

[23] Warburg MR Cohen N (1992b) Population dynamics, growth and longevity of *Armadillo officinalis* (Isopoda; Oniscidea), inhabiting the Mediterranean region of northern Israel. Pedobiologia 36: 262–273.

[24] Warburg MR Cohen N (1992c) Population structure, growth and longevity in two oniscid isopods. Proc. 4th ECE/ XIII, SEEC Gödöllő. Hungarian Natural History Musem, Budapest 2: 824–826

[25] Hornung E Warburg MR Szlavecz K (1992) Trends and methods in terrestrial isopod ecology: a round-table discussion. Proc. ECE/XIII, SEEC, Gödöllő. Hungarian Natural History Museum, Budapest 2: 747–750.

[26] Hornung E Warburg MR (1993) Breeding patterns in the oniscid isopod, *Porcellio ficulneus* Verh., at high temperature and under different photophases. Invertebrate Reproduction and Development 23: 151–158. doi: 10.1080/07924259.1993.9672306

[27] Warburg MR Cohen N Weinstein D Rosenberg M (1993) Life history of a semelparous oniscid isopod, *Schizidium tiberianum* Verhoeff, inhabiting the Mediterranean region of northern Israel. Israel Journal of Zoology 39: 79–93. doi: 10.1080/00212210.1993.10688698

[28] Warburg MR (1994a) Marsupial contents and losses due to putative intra-marsupial cannibalism by the mancas in three oniscid isopod species. Journal of Crustacean Biology 14: 560–67.

[29] Warburg MR (1994b) Review of recent studies on reproduction in terrestrial isopods. Invertebrate Reproduction and Development 26: 45–62. doi: 10.1080/07924259.1994.9672400

[30] Hornung E Warburg MR (1994) Oosorption and oocyte loss in *Porcellio ficulneus* B.-L. (Isopoda; Oniscidea; Porcellionidae) under stressful conditions. Tissue & Cell 26(2): 277–284. doi: 10.1016/0040-8166(94)90102-3 18621271 10.1016/0040-8166(94)90102-3

[31] Warburg MR (1995a) Continuous breeding in two rare, fossorial, oniscid isopod species from the Central Negev desert. Journal of Arid Environments 29: 383–93. doi: 10.1016/S0140-1963(05)80116-8

[32] Warburg MR (1995b) Growth and reproduction in a rare desert isopod: *Porcellio barroisi* (Oniscidea; Porcellionidae) from the Central Negev Mts. Journal of Arid Environments 31: 199–204. doi: 10.1006/jare.1995.0060

[33] Hornung E Warburg MR (1995a) Seasonal changes in the distribution and abundance of isopod species in different habitats within the Mediterranean region of northern Israel. Acta Oecologica 16: 431–45.

[34] Hornung E Warburg MR (1995b) Isopod distribution at different scaling levels. Crustacean Issues, 9 Balkema Publ. Rotterdam, Netherlands, 83–95.

[35] Heinzelmann F Crawford CS Warburg MR Molles MC (1995) Microhabitat selection of *Armadillidium vulgare* in a riparian forest: Lack of apparent influence by leaf litter food quality. Crustacean Issues, 9 Balkema Publ. Rotterdam, Netherlands, 133–143.

[36] Warburg MR Weinstein D (1995) Effect of temperature and photoperiod on the breeding pattern of two isopod species from the Mediterranean region of northern Israel. Balkema Publ. Rotterdam, Netherlands. Crustacean Issues 9: 107–119.

[37] Warburg MR Rosenberg M (1996) Brood-pouch structure in terrestrial Isopods. Invertebrate Reproduction and Development 29: 213–222. doi: 10.1080/07924259.1996.9672515

[38] Hornung E Warburg MR (1996) Intra habitat distribution of terrestrial isopods. European Journal of Soil Biology 32: 179–85.

[39] Warburg MR Adis J Rosenberg M Schaller F (1997) Ecology and the structure of respiratory organs in a unique amphibious/terrestrial philosciid isopod from the Neotropics. Studies on Neotropical Fauna & Environment 32: 52–63.

[40] Greenaway P Warburg MR (1998) Water fluxes in terrestrial isopods. Israel Journal of Zoology 44: 473–86. doi: 10.1080/00212210.1998.10688970

[41] Hornung E Warburg MR (1998) Plasticity of a *Porcellio ficulneus* population under extreme weather conditions (a case study). Israel Journal of Zoology 44: 395–8. doi: 10.1080/00212210.1998.10688961

[42] Warburg MR Hornung E (1999) Diversity of terrestrial isopod species along a transect through northern Israel. Biodiversity & Conservation 8: 1469–1478. doi: 10.1023/A:1008905729421

[43] Sharon R Degani G Warburg M (2001) Comparing soil macro-fauna in two oak-wood forests: does community structure differ under similar ambient conditions? Pedobiologia 45: 355–366. doi: 10.1078/0031-4056-00092

[44] Warburg MR Calahorra Y Amar KO (2001) Non-seasonal breeding in a porcellionid isopod. Journal of Crustacean Biology 21: 375–383. doi: 10.1651/0278-0372(2001)021[0375:nsbiap]2.0.co;2

[45] Warburg MR (2007) Patterns in the distribution, reproduction and abundance of the oniscid fauna of Israel. Crustaceana 80(10): 1223–1252. doi: 10.1163/156854007782321218

[46] Warburg MR (2011) Cost of breeding in oniscid isopods; a partial review. Crustaceana 84(12-13): 1561–1580. doi: 10.1163/156854011X607006

[47] Warburg MR (2012) The oniscid isopod female reproductive system and gestation, with a partial review. Invertebrate Reproduction and Development 56(2): 87–110. doi: 10.1080/07924259.2011.573812

[48] Warburg MR (2013) Post-parturial reproduction in terrestrial isopods: a partial review. Invertebrate Reproduction and Development 57(1): 10–26. doi: 10.1080/07924259.2011.633620

[49] Warburg MR (2013) Intra- and inter-specific variability in some aspects of the reproduction of oniscid isopods. Crustaceana 86(1): 98–109. doi: 10.1163/15685403-00003140

[B58] Hassall M Hornung E Warburg MR (Eds) (1998) Oniscidean Isopods. Proceedings of the 4th Symposium on the Biology of Terrestrial Isopods. Haifa. Israel Journal of Zoology 44: 1–250.

[B59] Warburg MR (1993) Evolutionary Biology of Land Isopods. Springer-Verlag, Berlin Heidelberg, 159 pp. doi: 10.1007/978-3-662-21889-1

